# A baseline cross-sectional survey of women’s and household-level health indicators in Tsakare Community, Mount Darwin District, Zimbabwe: findings to inform targeted public health interventions

**DOI:** 10.1080/16549716.2026.2746846

**Published:** 2026-10-16

**Authors:** Richard Makurumidze, Sibusiso Jokomo, Shuvai Chikombe, Memory Chimsimbe, Pamela Gorejena, Reason Tizora, Jayjay Karumazondo, Joseph Nyakuchena, Tinashe Mangena, George Mapiye, Cremance Tshuma, Rutendo Gutsire, Jephat Chifamba, Joconiah Chirenda, Fiona Makoni, Justen Manasa, Jonathan Matenga, Marvellous Mhloyi, Simbarashe Rusakaniko

**Affiliations:** a Faculty of Medicine and Health Sciences, University of Zimbabwe, Harare, Zimbabwe; b Faculty of Social and Behavioural Sciences, University of Zimbabwe, Harare, Zimbabwe; c Ministry of Health and Child Care, Harare, Zimbabwe

**Keywords:** Community health assessment, maternal and child health, HIV epidemiology, non-communicable diseases, communicable diseases, health service utilisation

## Abstract

**Background:**

A clear understanding of community health is essential for guiding public health interventions. This baseline survey was conducted within the University of Zimbabwe’s Education 5.0 initiative to generate foundational evidence for targeted interventions in the Tsakare Community, Mount Darwin District, Zimbabwe.

**Objective:**

To describe sociodemographic characteristics, maternal and child health, sexual and reproductive health, knowledge of hypertension and diabetes mellitus, HIV awareness, and household management of childhood illnesses among women in the Tsakare Community.

**Methods:**

A descriptive cross-sectional design was used, targeting women knowledgeable about household health issues. Participants were randomly selected and data collected via a structured questionnaire. Descriptive statistics were applied for analysis.

**Results:**

Data from 401 women were analysed. The median age was 31 years and median age at first marriage was 18 years. Hypertension symptom awareness was low, with only 41.7% recognising severe headaches as a symptom, while 17.1% had elevated blood pressure. For diabetes, 53.4% identified fatigue as a symptom, yet only 11.8% recognised genetic predisposition as a risk factor. HIV knowledge was moderate, with persistent misconceptions about non-risk behaviours. Most caregivers relied on clinic medication for childhood illnesses, though home and traditional remedies remained common.

**Conclusion:**

These findings highlight gaps in health literacy, early marriage practices, and limited awareness of chronic and infectious diseases in Tsakare. Targeted, Education 5.0 driven interventions are needed to improve health literacy, strengthen maternal and child health services, and scale up prevention and screening for chronic and infectious diseases.

## Paper Context

- **Main findings:** While there is high utilization of formal healthcare for childbirth, the community exhibits significant gaps in health literacy regarding non-communicable diseases, with low awareness of symptoms for hypertension and diabetes despite 17.1% of participants recording elevated blood pressure.
- **Added knowledge:** This research provides the foundational baseline data required for the development of targeted public health interventions in rural Zimbabwe, specifically documenting the intersection of early marriage practices, moderate HIV knowledge, and the coexistence of traditional and modern healthcare-seeking behaviours.
- **Global health impact for policy and action:** These findings serve as a framework for policymakers to design culturally sensitive, community-led surveillance structures and targeted educational interventions that optimize resource allocation and accelerate progress toward Sustainable Development Goals in low-resource settings.

## Background

Understanding the general health status of a community is paramount for informing effective public health interventions and policy decisions [1]. As part of the University of Zimbabwe Education 5.0 implementation, this study serves as a baseline survey aimed at assessing the health issues prevalent within the Tsakare Community, Mount Darwin District, Zimbabwe [2]. By collecting comprehensive data on various health indicators, this study aims to provide a foundational understanding that can guide tailored interventions to enhance overall community well-being. A thorough assessment of health issues is essential before implementing targeted interventions [2,3]. Such an understanding allows public health officials and policymakers to identify specific health challenges faced by the community, including maternal health, chronic diseases, and infectious diseases [4].

The University of Zimbabwe, through its Education 5.0 inter-faculty initiative, prioritised Tsakare Community for community-engaged research, with the Faculty of Medicine and Health Sciences tasked to address key public health challenges. Tsakare, a predominantly rural community in Ward 36 of Mount Darwin District in northern Zimbabwe, faces typical constraints of remote settings, including limited access to health services, low household incomes, and geographic barriers to healthcare. Education 5.0 marks a shift from the more inward-looking Education 3.0 model by requiring universities to form direct, solution-oriented partnerships with communities, integrating teaching, research, community engagement, innovation, and industrialisation to tackle complex health problems such as the emerging syndemic in Tsakare Community. It emphasises communication, participation, and co-creation, ensuring that interventions are both evidence-based and culturally relevant [5]. Within this framework, the present women-focused descriptive baseline survey was undertaken to generate foundational evidence for targeted public health interventions, developed in collaboration with local stakeholders, including community members and healthcare providers.

For instance, maternal health is a significant component of public health that reflects broader societal conditions, including education levels, economic stability, and access to healthcare services. The World Health Organization (WHO) emphasizes that improving maternal health outcomes is vital for achieving Sustainable Development Goals, particularly in low- and middle-income countries where maternal mortality rates remain high [6,7]. In addition to maternal health, awareness of chronic diseases such as hypertension and diabetes mellitus is increasingly recognized as vital for public health. Hypertension is often referred to as the ‘silent killer’ due to its asymptomatic nature until severe complications arise [8]. Similarly, awareness surrounding diabetes remains insufficient and this lack of awareness can lead to late diagnoses and increased morbidity and mortality associated with these chronic conditions [9]. Moreover, understanding the awareness of infectious diseases such as HIV is critical in preventing transmission and improving community health outcomes. Knowledge about how HIV is transmitted, and available prevention strategies can significantly reduce infection rates. However, many communities still grapple with stigma and misinformation surrounding HIV, which can hinder effective prevention efforts [10]. Management of common childhood illnesses, including fever, diarrhoea, and cough, is another important dimension of community health, as treatment choices and care seeking patterns directly influence child morbidity and mortality.

The rationale for this baseline survey was the need to generate foundational evidence for targeted public health interventions in the area. Developing meaningful interventions requires a clear understanding of existing health conditions, knowledge gaps, and barriers to healthcare access within the population. Such evidence supports the design of tailored interventions and monitoring mechanisms that respond to community-specific needs. For example, identifying limited awareness of hypertension symptoms can inform the integration of targeted health education and screening into the surveillance framework, thereby improving prevention and early detection of chronic diseases.

By first establishing a comprehensive picture of the community’s health profile, monitoring and evaluation systems can be put in place to track key indicators over time and support evidence-based decision-making. In line with national public health priorities in Zimbabwe, where the dual burden of communicable and non-communicable diseases account for a substantial share of morbidity and mortality, hypertension, diabetes mellitus, HIV, maternal and child health, sexual and reproductive health, and management of common childhood illnesses were selected as focal domains for this baseline survey so that the findings would directly inform high‑impact, community‑based interventions across the major drivers of health in Tsakare Community.

The specific objectives were to describe the sociodemographic characteristics of women in the community, examine maternal and child health and sexual and reproductive health, evaluate knowledge of common non-communicable diseases such as hypertension and diabetes mellitus, explore knowledge of HIV transmission and prevention, and describe household management practices for common childhood illnesses. Addressing challenges in these domains is essential for strengthening population health monitoring, guiding resource allocation, and advancing progress toward national and global Sustainable Development Goals, particularly those related to maternal, child, and reproductive health.

## Methodology

### Study design

This study employed a quantitative descriptive cross-sectional design to assess knowledge on selected health conditions, maternal health practices, and sociodemographic characteristics among women in Tsakare Community, located in the Mount Darwin District of Zimbabwe. All data were collected through structured self-reporting, except for blood pressure, which was directly measured. Complementary qualitative data were also collected to enrich the quantitative findings; however, these were analysed and reported separately in another publication [11]. The study was conducted in accordance with the ethical principles of the Declaration of Helsinki. Ethical approval was obtained from the Medical Research Council of Zimbabwe (MRCZ/A/3191). Additional permissions were obtained from the relevant local and district authorities.

### Study setting

The research was conducted in Tsakare Community, situated in Ward 36 of Mount Darwin District. Mount Darwin District is a second-tier administrative unit in Mashonaland Central Province in northern Zimbabwe. It is predominantly rural, and communities depend largely on subsistence farming, smallholder agriculture, informal trade, and some mining activities. Tsakare Community, administered under traditional leadership and Pfura Rural District Council, comprises several villages, which represent the fourth administrative tier, with households clustered in low-lying areas. Health services in the district are provided through a network of rural health centres, clinics, and one district hospital, with Tsakare Clinic serving as the community’s primary health facility. Limited staffing, equipment, and referral capacity at the clinic constrain access to care and contribute to service-delivery challenges. Access to secondary and tertiary education is also limited, with only a small number of primary and secondary schools in the area. Basic infrastructure includes gravel roads, boreholes for domestic water, and mobile-phone coverage, whereas formal health, financial, and recreational facilities remain scarce. Households therefore often face geographic barriers to healthcare access.

The Tsakare Community was prioritised for Education 5.0 because it embodies many of the complex, interlinked health and development challenges that the framework seeks to address, including high burdens of preventable disease, resource constraints, and structural barriers to care. At the same time, its strong traditional leadership, existing community laboratory, and demonstrated willingness to engage with the University of Zimbabwe provide a conducive environment for co-created transdisciplinary interventions that integrate all Education 5.0 elements i.e. teaching, research, community engagement, innovation and industrialization.

### Study population

The study targeted women from the sampled households within the Tsakare Community who were knowledgeable about household issues. This criterion was established to ensure that respondents could provide accurate and relevant information regarding their household health dynamics and awareness of health issues. Women were selected as respondents because of their central involvement in household caregiving and health-related reporting. We acknowledge, however, that health decision-making may be shared between partners within households. Households without an eligible adult woman at the time of survey implementation were not included, which may have introduced selection bias. Where more than one eligible woman resided in a selected household, one respondent was selected using a simple random approach to ensure one interview per household.

### Sample size

The sample size for this study was calculated using Dobson’s formula for cross-sectional studies. The calculation assumed a Z-score of 1.96 for a 95% confidence level, a margin of error of 5% (0.05), and an expected proportion of 50%. The 50% proportion was selected because it yields maximum variability and therefore the largest required sample when estimating multiple health indicators. These parameters produced the required sample size of 385 participants. This sample size was intended to capture demographic variation across households and support the application of the findings to the broader community. Including participants from different households also allowed variation in the health awareness and behaviours to be described, increasing the usefulness of the findings for public health interventions.

### Sampling procedure

A random sampling procedure was used to select participating households. A household sampling frame was compiled prior to fieldwork using a community household list obtained from local administrative records. Households were then selected using systematic random sampling, whereby every nth household on the list was chosen after a random start.

This approach ensured that every household had an equal probability of selection, minimizing selection bias and enhancing the representativeness of the sample.

### Study variables

The study examined several domains to provide a comprehensive picture of health in the Tsakare Community. Participant‑level variables included sociodemographic characteristics such as age, marital status, educational attainment, and age at first marriage. Household‑level variables reported by women covered household size, number of rooms, and number of sleeping rooms. Maternal and child health and sexual and reproductive health indicators were assessed to understand patterns of marriage, pregnancy, and use of formal maternity care. The survey also explored knowledge of hypertension and diabetes mellitus, focusing on recognition of symptoms, awareness of risk factors, and understanding of prevention measures. Knowledge of HIV transmission routes and prevention strategies was examined, particularly in relation to common misconceptions and awareness of proven protective behaviours. Finally, the study documented household management practices for common childhood illnesses, including fever, diarrhoea, and cough, to capture everyday care seeking and treatment patterns in the community.

### Data collection tools and procedures

Data were collected using Open Data Kit (ODK), a mobile platform that supports efficient and accurate data entry in field settings. Trained enumerators administered the structured questionnaires to ensure consistency. Interviews were conducted predominantly in Shona, the local language spoken in Tsakare Community, and the questionnaire was translated before fieldwork to improve linguistic accessibility and response consistency. Enumerators were also trained to measure blood pressure using a digital sphygmomanometer. Each participant was seated, and the cuff was placed on the bare upper arm; the device then inflated and deflated automatically while calculating systolic and diastolic blood pressure using the oscillometric method. Owing to logistical constraints, only one measurement was obtained per participant. The blood pressure findings should therefore be interpreted cautiously because a single reading may misclassify blood pressure status compared with repeated-measurement protocols. All other health-related variables, including self-reported hypertension or diabetes status and knowledge of disease symptoms, risk factors, and prevention measures, were obtained through participant self-report.

### Data analysis

Data analysis was conducted using Stata 17 statistical software. Descriptive statistics were employed to summarize the sociodemographic characteristics of participants and their knowledge of health issues. Frequencies and percentages were calculated for categorical variables, whereas medians and ranges were calculated for continuous variables. The results are presented in tables and figures for clarity. High blood pressure was defined as either a systolic blood pressure of ≥140 mm Hg or a diastolic blood pressure of ≥90 mm Hg, based on the single reading obtained during the survey interview. This classification was based solely on the measured reading and did not incorporate current antihypertensive treatment status, which is acknowledged as a limitation.

## Results

### Sociodemographic characteristics

Data from 401 women were analysed. Because some variables had item-specific missing responses, denominators varied across analyses; percentages were calculated from the available responses and are reported with the relevant denominators in each table. The median participant age was 31 years (Q1: 23; Q3: 43). Most participants were married (81.5%), whereas 6.0% were divorced or separated and 10.7% were widowed. Educational attainment was generally low: 56.9% had completed primary education, 0.7% had attained an advanced level, and 4.2% (17 of 401) had never attended school. The median household size was four people (range: 1–12), and the median number of rooms per household was three (range: 1–9) (Table 1).

**Table 1.** Sociodemographic characteristics of women in Tsakare Community.

Variable (n)	Category	Frequency, Median or Range	Percentages
Age (399)	Median (Q1, Q3)	31 (23, 43)	
	Range (min, max)	16, 83	
Marital status (401)	Never Married	5	1.2
	Married	327	81.5
	Divorced/Separated	24	6.0
	Widowed	43	10.7
	Missing	2	0.5
Highest level (401)	Never	17	4.2
	Primary	228	56.9
	Ordinary	150	37.4
	Advanced	3	0.7
	Missing	3	0.7
Number of people in household (399)	Median (Q1, Q3)	4 (3, 6)	
	Range (min, max)	1, 12	
Number of Rooms at household (397)	Median (Q1, Q3)	3 (2, 4)	
	Range (min, max)	1, 9	
Sleeping Rooms (397)	Median (Q1, Q3)	2 (1, 2)	
	Range (min, max)	1, 7	

### Maternal sexual and reproductive health

Table 2 presents data on maternal and reproductive health. The denominators in Table 2 vary across variables and reflect the eligible subgroup for each item. Specifically, the 397 respondents with data on age at first marriage include all women who had ever been married, which exceeds the 327 currently married women reported in Table 1. The median age at first marriage among participants was 18 years, with a range from 16 to 20 years. Among currently married women (*n* = 327), 67.3% were in monogamous marriages and 32.7% were in polygamous marriages. The median age of spouses was reported at 36 years (Q1:28, Q3:45), with notable age differences observed – some men being significantly older than their partners. The median age of sexual debut was also recorded at 18 years. Attendance at births was predominantly by healthcare professionals (77.4%), while traditional birth attendants were involved in 10.8% of cases. Among women with data on antenatal care (ANC) attendance during their most recent pregnancy, 77.0% of participants attended at least one antenatal care visit during their last pregnancy.

**Table 2.** Women’s sexual and reproductive health in Tsakare Community.

Variable	Category	Median, frequency or range	Percentage
Age at first marriage (397)	Median (Q1, Q3)	18 (16, 20)	
	Range (min, max)	13, 30	
Sequence in marriage (107)	Range (min, max)	1, 9	
Type of marriage (327)	Monogamous	220	67.3
	Polygamous	107	32.7
Age of spouse (399)	Median (Q1, Q3)	36 (28, 45)	
	Range (min, max)	19, 91	
Age difference (range)	Man older	0, 63	
	Woman older	1, 17	
Age of sexual debut (390)	Median	18 (13, 28)	
	Range (min, max)	12, 40	
Who attended last birth (371)	Family member	31	8.4
	Healthcare worker	287	77.4
	Other	11	3.0
	Self	1	0.3
	Traditional birth attendant	40	10.8
Attended at least 1 ANC visit last pregnancy (369)	No	85	23.0
	Yes	284	77.0

### Knowledge of hypertension: symptoms and prevention measures

The study assessed knowledge regarding hypertension symptoms and preventive measures among participants. A notable 41.7% recognized severe headaches as a symptom, while other common symptoms included chest pains (32.5%) and dizziness (34.4%). Blood pressure measurements indicated that 61 of 357 measured participants (17.1%) had a high blood pressure. Knowledge of preventive strategies was limited; getting adequate sleep was recognized by 34.6%, maintaining healthy weight by 22.9%, and exercise by only 15.4% (Table 3).

**Table 3.** Women’s knowledge of hypertension symptoms and prevention measures in Tsakare Community.

Variable (n)	Categories	Frequency	Percentage
Awareness of symptoms (381)	Severe headaches	159	41.7
	Chest pains	124	32.5
	Dizziness	131	34.4
	Difficulty breathing	115	30.2
	Blurred vision	49	12.9
	Anxiety	65	17.1
	Nausea	60	15.7
	Vomiting	44	11.5
Measured Blood Pressure (357)	High	61	17.1
	Normal	296	82.9
Awareness of prevention measures (376)	Exercise	58	15.4
	Physical activity	74	19.7
	No smoking	41	10.9
	Maintaining a healthy diet	84	22.3
	Maintaining a healthy weight	86	22.9
	Limiting alcohol intake	55	14.6
	Getting enough sleep	130	34.6

### Knowledge of diabetes mellitus: symptoms and prevention measures

The findings on diabetes mellitus awareness revealed that fatigue was the most recognized symptom, reported by 53.4% of participants, followed by increased thirst (41.9%) and frequent urination (26.7%). Knowledge of the three cardinal symptoms of diabetes, frequent urination, increased hunger, and increased thirst, was low among participants. Regarding risk factors, 37.9% identified consumption of foods high in sugar, whereas only 11.8% recognised genetic predisposition. Knowledge of diabetes prevention was similarly limited. Limiting consumption of foods high in sugar was reported by 41.6% of participants, limiting red meat consumption by 16.2%, and regular exercise by only 12.6% (Table 4).

**Table 4.** Women’s knowledge of diabetes mellitus symptoms, risk factors, and prevention measures in Tsakare Community.

Variables	Categories	Frequency	Percentage
Awareness of symptoms (191)	Frequent urination	51	26.7
	Increased thirst	80	41.9
	Abnormal hunger	64	33.5
	Fatigue	102	53.4
	Weight loss	38	19.9
	Blurred vision	52	27.2
Awareness of risk factors (380)	Genetic	45	11.8
	overweight	74	19.5
	Physical inactivity	85	22.4
	Foods high in sugar	144	37.9
Awareness of prevention measures (382)	Eating natural foods	81	21.2
	Exercise	48	12.6
	Limiting high sugar foods	159	41.6
	Limiting red meat	62	16.2

### Knowledge of HIV transmission and prevention measures

Most participants correctly identified behaviours that do not transmit HIV. Specifically, 78% correctly stated that sharing food with a person living with HIV does not transmit the virus, while 77% correctly stated that mosquito bites do not transmit HIV. However, 9% believed that witchcraft or supernatural means could transmit HIV. Awareness of mother to child transmission was higher, with 68% recognising the risk during breastfeeding and 77% understanding the risk during pregnancy.

Knowledge of prevention strategies was variable. Notably, 78% held the misconception that a circumcised man can engage in sexual intercourse without a condom without increasing his or his partner’s risk of HIV. In contrast, approximately 79% recognised that having one uninfected partner reduces the risk of HIV infection (Figure 1).

**Figure 1.**
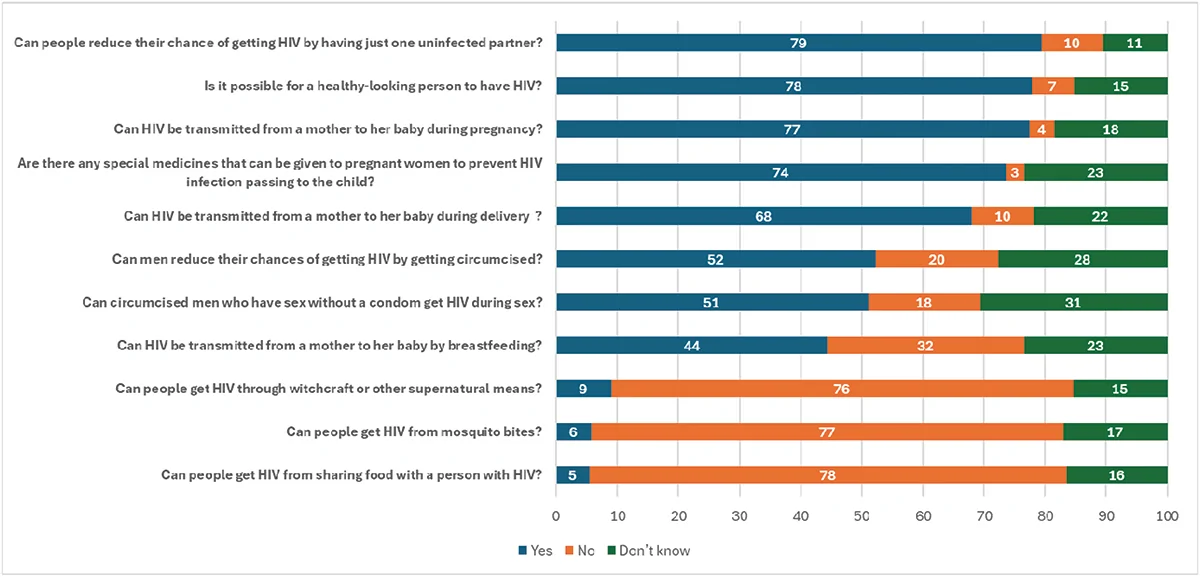
Women’s knowledge of HIV transmission and prevention in Tsakare Community.

### Management practices of common childhood illnesses

The study examined the approaches used by women to manage common childhood illnesses, including fever, diarrhoea, and cough. The findings showed that caregivers used a range of treatment approaches, including clinic medication, home remedies, spiritual treatments, traditional treatments, and combinations of these approaches.

For fever, most caregivers (59.6%) relied solely on medication from clinics. Home remedies were used by 15.8%, while 5.3% combined home remedies with clinic medication and 5.3% combined home remedies with spiritual treatments. A smaller proportion used spiritual treatments alone (1.8%) or a combination of spiritual treatments and home remedies (1.8%).

For diarrhoea, 61.9% of caregivers used medication from clinics. Home remedies were used by 14.3%, while 6.3% used a combination of home remedies and clinic medication. Spiritual treatments alone were used by 1.6%, whereas the combination of spiritual treatments and home remedies was not significantly reported.

For cough, treatment approaches were more varied. Most caregivers (63.8%) used medication from clinics, while 8.6% used home remedies. A combination of home remedies and spiritual treatments was reported by 5.2%, whereas 3.4% combined clinic medication with home remedies. Spiritual treatments alone were used by 6.9%, and 1.7% used a combination of spiritual treatments and home remedies. In addition, 10.3% of caregivers used traditional treatments (Figure 2).

**Figure 2.**
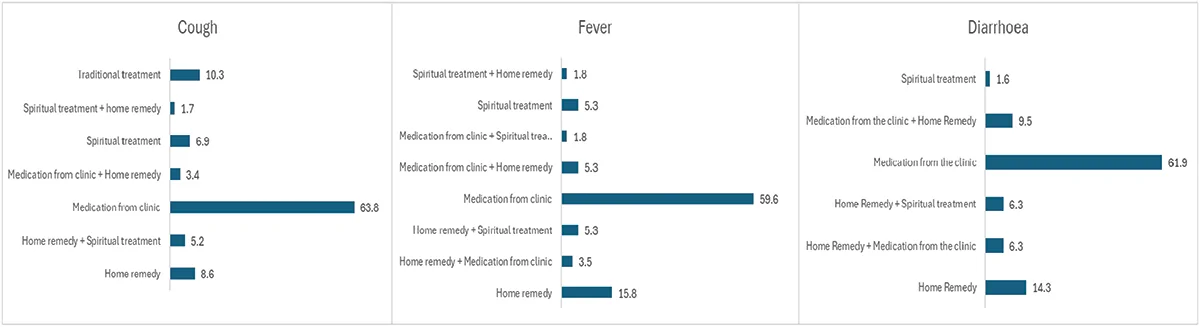
Management of cough, fever and diarrhoea.

## Discussion

This baseline study in Tsakare Community provides evidence to inform targeted public health interventions. The findings describe important patterns in sociodemographic characteristics, maternal and reproductive health, knowledge of chronic and infectious diseases, and household management of common childhood illnesses. Educational attainment was generally low, with most participants having completed only primary education. The median age at first marriage was 18 years, and most reported births were attended by healthcare professionals. Knowledge of hypertension and diabetes symptoms was limited, and preventive measures were poorly recognised. There was co-existence of clinic-based, home, spiritual, and traditional approaches in the management of childhood illnesses.

The sociodemographic profile of the participants indicates a young population, which has significant implications for public health interventions. The predominance of married individuals suggests a stable family structure that can facilitate health education dissemination within households. The high percentage of married individuals (81.5%) is consistent with cultural norms in many African communities, where early marriage is common [12,13]. For instance, a study in Malawi reported that approximately 75% of women were married by age 25 [14]. This similarity underscores the importance of tailoring health interventions to the family structure prevalent in these communities. The median household size of four members is consistent with typical rural household sizes in Southern Africa and should not automatically be interpreted as indicative of overcrowding. Overcrowding assessments require additional information such as the number of persons per sleeping room and household composition by age and sex, which were partially available through sleeping room data in the present survey.

Low educational attainment raises concerns about health literacy, which is important for understanding health risks and preventive measures. This finding is consistent with studies from rural African settings, where educational barriers affect health literacy and access to healthcare services [15,16]. Previous research has associated higher educational attainment with better health outcomes through improved access to information and resources [17,18]. Limited education may therefore reduce individuals’ ability to make informed health decisions and engage with available services. For example, a study in Ethiopia found that women with secondary education had greater awareness of reproductive health issues [19]. These findings highlight the need for educational programmes that reflect the community’s context and cultural norms. Future public health interventions in Tsakare Community should address low literacy through interviewer-administered tools, local-language communication, visual aids, and sustained engagement with community leaders and village health workers.

The findings related to maternal, sexual and reproductive health reveal critical insights into early marriage practices and healthcare utilization during childbirth. The median age at first marriage being 18 years aligns with trends observed in other regions where early marriage is prevalent [12,13]. This practice can have profound implications for women’s health, as early pregnancies are often associated with higher risks of complications [20]. The Tsakare Community study indicates that 32.7% of marriages were polygamous. This prevalence is consistent with findings from other rural communities where polygamous unions are culturally accepted [21]. In contrast, urban studies have reported substantially lower levels of polygamy [22]. The practice of polygamy may reflect underlying cultural and religious beliefs within specific communities.

The reliance on healthcare professionals for childbirth (77.4%) indicates a positive trend towards formal healthcare utilization [23]; however, the involvement of traditional birth attendants (10.8%) suggests that traditional practices still hold sway in the community [24]. This duality necessitates culturally sensitive approaches in public health campaigns that respect traditional beliefs while promoting modern healthcare benefits. Furthermore, the high attendance rate at antenatal care visits (77%) is encouraging but highlights the need for continued efforts to ensure that all women receive comprehensive prenatal care [25].

Knowledge of hypertension symptoms was moderate, with only 41.7% of participants recognising severe headaches as a symptom. This finding is consistent with studies conducted in rural settings, where awareness of hypertension symptoms remains low [26]. This knowledge gap is important because 17.1% of participants with available measurements had elevated blood pressure based on a single reading; however, this result does not establish the prevalence of hypertension or indicate how many participants were unaware of their blood pressure status. A study in Tanzania similarly reported that fewer than 10% of respondents could identify common symptoms associated with high blood pressure [27]. Limited awareness is concerning given the growing global burden of hypertension and its association with cardiovascular diseases [28]. Recognition of preventive strategies was also low, including exercise (15.4%). Studies in urban settings have reported greater awareness of hypertension symptoms and prevention methods [26], possibly reflecting differences in access to health information and services. Lifestyle-focused interventions in rural communities should recognise that activities such as walking long distances, farming, and fetching water may already involve substantial physical exertion. Interventions should therefore combine context-appropriate physical activity guidance with dietary modification and targeted reduction of other risk factors.

The finding that 17.1% of measured participants in Tsakare Community had elevated blood pressure indicates a potentially important public health concern. Because blood pressure was measured only once and treatment status was not incorporated into the classification, this proportion should not be interpreted as the prevalence of clinically diagnosed hypertension. Nevertheless, elevated blood pressure is an important risk marker because sustained hypertension is associated with cardiovascular disease, stroke, and kidney failure [8]. Globally, approximately 1.13 billion people have been estimated to have hypertension, many of whom are unaware of their condition [29]. The limited recognition of hypertension symptoms and preventive measures in Tsakare Community supports the need for health education and repeated blood pressure screening. Regular screening activities conducted with local healthcare providers could improve early detection, referral, and management [30].

The study found that fatigue was the most recognized symptom of diabetes mellitus among participants (53.4%), yet knowledge of risk factors remained low, particularly regarding genetic predisposition (11.8%). The finding that knowledge of the three cardinal signs of diabetes – frequent urination, abnormal hunger, and increased thirst – was low among participants in the Tsakare Community is also concerning. This aligns with findings from other studies indicating that knowledge about diabetes risk factors is often inadequate in rural populations [31,32]. For example, a study conducted in Mali found that knowledge of diabetes symptoms was alarmingly low among rural populations, with many individuals unable to identify common signs such as increased thirst and frequent urination [33]. Similarly, a study in Nigeria reported that only 34.1% of participants recognized the cardinal symptoms of diabetes [34]. The recognition of limiting high sugar foods as a preventive measure by 41.6% is promising; however, the low emphasis on exercise (12.6%) points to an area needing urgent attention in public health messaging. Community-based programs promoting physical activity alongside dietary changes could foster better understanding and management of diabetes risks.

Knowledge regarding HIV transmission modes revealed significant misconceptions among participants. While many recognized mother-to-child transmission during pregnancy and breastfeeding (68% and 77%, respectively), misunderstandings about non-risky behaviours persisted, with 5% believing that sharing food could transmit HIV [35]. This disparity highlights the need for comprehensive education campaigns addressing myths surrounding HIV transmission while reinforcing correct information about prevention strategies. The strong awareness regarding circumcision as a preventive measure suggests that culturally appropriate interventions could leverage existing knowledge to enhance understanding further [36].

The management practices for common childhood illnesses such as fever, diarrhoea, and cough demonstrated a reliance on clinic medications (59.6% for fever) but also highlighted the use of home remedies (15.8%). This blend reflects a common trend in many communities where traditional beliefs co-exist with modern medical practices [37–39]. However, the limited use of spiritual treatments indicates a shift towards more evidence-based approaches to healthcare among the caregivers. Public health initiatives should aim to educate caregivers about effective treatments while respecting their cultural practices to improve childhood illness management [40].

### Strengths and limitations

This study has several strengths and limitations that should inform interpretation of the findings. Its comprehensive data-collection tool covered multiple health domains and provided a broad description of health issues in Tsakare Community. The inclusion of several sociodemographic variables also supported examination of factors that may influence health knowledge and behaviours. In addition, the cross-sectional design provided a snapshot of community health at a defined point in time, establishing baseline information for the design and evaluation of future interventions.

However, the study also has several limitations. The reliance on self-reported data may introduce bias, as participants could underreport or overreport their health behaviours and awareness levels. Relatively low educational attainment among many participants may have affected their ability to fully understand some questions and to accurately report health issues, potentially skewing the results. The focus on women in households with an eligible respondent, and the exclusion of households without an adult woman, may have introduced selection bias and limits representativeness of the wider community. Important demographic and household condition variables, such as full fertility and mortality histories, migration patterns, housing materials, water and sanitation, and household assets, were not collected, restricting the ability to generate core demographic indices or examine wealth‑related gradients in health. Blood pressure measurements were taken only once per participant using a digital device, and current antihypertensive treatment status was not recorded, so estimates of elevated blood pressure may be affected by measurement error and misclassification. Finally, although the overall sample size was adequate for descriptive purposes, item‑level non-response reduced denominators for several variables and the modest sample limited detailed subgroup analyses, which may affect the generalisability of some of the findings to the broader Tsakare Community population and similar rural settings.

### Recommendations

The findings from this baseline study provide critical evidence to guide targeted public health interventions in the Tsakare Community within the University of Zimbabwe’s Education 5.0 framework. The University, policymakers, and health authorities should use these results to design Education 5.0‑driven activities that regularly monitor key health domains, including demographic patterns, maternal and child health, sexual and reproductive health (such as early marriage), and the burden of communicable and non-communicable diseases.

Strengthening community health education and literacy should be a core Education 5.0 priority, with multidisciplinary teams of students and staff leading locally tailored campaigns on hypertension, diabetes, HIV, and childhood illnesses. These outreach efforts should specifically address low awareness of chronic disease symptoms and risk factors, as well as persistent HIV related misconceptions and stigma. Routine, household-based data collection embedded in community engaged teaching and research will allow continuous tracking of health indicators and improve responsiveness of interventions.

Health service delivery initiatives should build on existing patterns of clinic use by expanding preventive and screening services for hypertension and diabetes through community health workers and outreach from Tsakare Clinic. Collaborations between medical, public health, engineering, social sciences and business faculties can support improvements in clinic infrastructure and basic equipment, strengthen referral pathways, and co-design simple, sustainable models for implementing evidence-based community health initiatives. Culturally sensitive engagement strategies, developed with traditional leaders and Pfura Rural District Council, are essential to ensure community ownership and acceptance of Education 5.0 aligned programmes.

Future research under Education 5.0 should also address key limitations of this baseline survey. Larger, follow up studies should include men as well as women, collect fuller demographic and household condition data (fertility, mortality, migration, housing and assets), and use repeated clinical measurements to better estimate the burden and control of non-communicable diseases. A follow up study focused on hypertension and diabetes is needed to establish robust baseline prevalence, understand treatment and control patterns, and evaluate the impact of targeted education and screening interventions. Longitudinal or repeated cross sectional data collection will enable trend monitoring and strengthen the capacity of Education 5.0 driven community university partnerships to prevent and manage chronic and infectious diseases in Tsakare Community and similar rural settings.

## Conclusion

In conclusion, this baseline cross-sectional survey in Tsakare Community demonstrates interlinked health challenges and actionable opportunities that can inform Education 5.0-aligned public health interventions. The results show limited awareness of and engagement in preventive behaviours for chronic diseases, notably hypertension and diabetes, alongside early marriage, constrained maternal and child health services, and persistent misconceptions about HIV transmission and prevention. Household management of childhood illnesses combines clinic-based care with home and traditional remedies, underscoring the need for culturally sensitive, evidence-informed health education. By using these findings to strengthen community health literacy, expand prevention and screening services through Tsakare Clinic and community health workers, and improve basic health infrastructure, university–community partnerships can help improve health outcomes in this rural setting. Larger and more comprehensive studies that include men, collect richer demographic and household data, and use repeated clinical measurements will be important for improving estimates of disease burden and guiding the design and evaluation of sustained Education 5.0-driven interventions in Tsakare and similar communities.

## Supplementary Material

STROBE Checklist.docx

## Data Availability

The data will be made available upon reasonable request to the corresponding author.
